# Malaria misdiagnosis in the routine health system in Arba Minch area district in southwest Ethiopia: an implication for malaria control and elimination

**DOI:** 10.1186/s12936-023-04711-2

**Published:** 2023-09-14

**Authors:** Engida Yigezu, Biniam Wondale, Daniel Abebe, Girum Tamiru, Nigatu Eligo, Bernt Lindtjørn, Endalamaw Gadisa, Fitsum Girma Tadesse, Fekadu Massebo

**Affiliations:** 1 Department of Biology, Arba Minch University, Arba Minch, Ethiopia; 2 Institute of Biotechnology, Addis Ababa University, Addis Ababa, Ethiopia; 3 Armauer Hansen Research Institute, Addis Ababa, Ethiopia; 4 Centre for International Health, University of Bergen, Bergen, Norway

**Keywords:** Health facility, Malaria misdiagnosis, Microscopic diagnosis, Mixed infection

## Abstract

**Background:**

*Plasmodium falciparum* and *Plasmodium vivax* are coendemic in Ethiopia, with different proportion in different settings. Microscopy is the diagnostic tool in Ethiopian health centres. Accurate species-specific diagnosis is vital for appropriate treatment of cases to interrupt its transmission. Therefore, this study assessed the status of species-specific misdiagnosis by microscope compared with polymerase chain reaction (PCR).

**Methods:**

A health facility based cross-sectional study was conducted from November 2019 to January 2020 in Kolla Shelle Health centre, Arba Minch Zuria district. The study population were suspected malaria cases, who visited the health centre for a diagnosis and treatment. Consecutive microscopy positive cases as well as a sample of microscopically negative cases were included for molecular analysis by polymerase chain reaction (PCR).

**Results:**

254 microscopically negative and 193 microscopically positive malaria suspects were included. Of the 193 malaria positive cases, 46.1% [95% confidence interval (CI) 38.9–53.4] (89/193) were *P. falciparum* infection, 52.3% (95% CI 45.0–59.5) (101/193) were *P. vivax* infection, and 1.6% (3/193) had mixed infection of *P. falciparum* and *P. vivax*. Of the microscopically positive cases of *P. falciparum*, 3.4% (3/89) were *P. vivax* and 11.2% (10/89) were mixed infections with *P. falciparum* and *P. vivax* and a single case was negative molecularly. Similarly, of the microscopically positive *P. vivax* cases, 5.9% (6/101) were *P. falciparum* and 1% (1/101) was mixed infection. Single case was negative by molecular technique. Of the 254 microscopically negative cases, 0.8% were tested positive for *P. falciparum* and 2% for *P. vivax* by PCR*.* Considering molecular technique as a reference, the sensitivity of microscopy for detecting *P. falciparum* was 89.2% and for *P. vivax*, it was 91.2%. The specificity of microscopy for detecting *P. falciparum* was 96.1% and for *P. vivax*, it was 97.7%. However, the sensitivity of microscopy in detecting mixed infection of *P. falciparum* and *P. vivax* was low (8.3%).

**Conclusion:**

There were cases left untreated or inappropriately treated due to the species misidentification. Therefore, to minimize this problem, the gaps in the microscopic-based malaria diagnosis should be identified. It is recommended to regularly monitor the competency of malaria microscopists in the study area to improve species identification and diagnosis accuracy.

## Background

Malaria is a vector borne diseases caused by *Plasmodium* parasite. In 2020, there were about 241 million malaria cases globally. Of which 96% of cases were reported from 29 malaria endemic countries [1]. Africa contributed for 95% of global malaria cases and 96% deaths. The high burden six countries in Africa contributed 55% of all malaria cases and 54% of malaria related deaths in sub-Saharan Africa [1]. Despite the fact that *Plasmodium falciparum* and *Plasmodium vivax* are the two most common parasite species, *Plasmodium ovale*, *Plasmodium malariae* and *Plasmodium knowlesi* are increasingly responsible for malaria-related illnesses and deaths [2]*.*

Among the methods available for malaria diagnosis, microscopy, rapid diagnostic test (RDT), polymerase chain reaction (PCR), and loop-mediated isothermal amplification (LAMP) are a few of the most common. Each diagnostic technique has its own advantages and limitations based on accuracy, cost, availability of trained personnel, and infrastructure [3]. Microscopy is widely used to detect malaria parasites [4], but its effectiveness depends on laboratory technicians’ skills and parasite density. In areas with low parasitaemia or mixed infections, nested PCR is thought to improve malaria diagnosis [5, 6]. Anti-malarial drug resistance can be reduced and patient outcomes improved with accurate parasite identification [5, 7].

*Plasmodium falciparum* and *P. vivax* coexist in Ethiopia with 60% *P. falciparum* and 40% *P. vivax* [1]. Accurate detection of the two parasites is crucial in malaria control and elimination programmes as it saves money, prevents treatment delays and poor patient outcomes, and reduces the likelihood of drug resistance [4]. An erroneous diagnosis might result in an inappropriate course of action and further case complications [8, 9]. In Ethiopia’s health centres, microscopy is the primary method for diagnosing malaria. However, it is crucial to verify these results using more sensitive techniques to achieve the country’s aim of controlling and eliminating malaria by 2030. Hence, the objective of this study was to validate the microscopic results in samples from a health facility in Arba Minch district, southwest Ethiopia using a more sensitive molecular technique.

## Methods

### Study area description

This study was conducted in Kolla Shelle Health Centre, Arba Minch Zuria district of Gamo zone, southwest Ethiopia. Arba Minch town serves as the district's hub. There are 18 *Kebeles* (the lowest administrative unit) in the district. Ten are in the lowlands, and eight are in the middle lands. Kolla Shelle is one of the *Kebeles* where malaria is endemic. Kolla Shelle Health Centre serves six *Kebeles* in the catchment. It also supports 11 health posts within the catchment, serving for about 47,970 inhabitants. *Anopheles arabiensis* is the primary malaria vector whereas *Anopheles pharoensis* plays a secondary role [10, 11].

### Study design, study population and sample size

A health facility based cross-sectional study was conducted from November 2019 to January 2020. The study population was individuals with suspected (with fever, headaches, and other signs and symptoms of malaria are taken into account) malaria aged five or older who visited the study health centre for malaria diagnosis and treatment during the study period. Consecutive positive cases as well as a sub-sample of microscopically negative cases were included. This study aimed to compare nested nPCR to microscopy in order to determine the percentage of malaria cases that were incorrectly diagnosed or overlooked by microscopy. Therefore, the sample size that was included in this time-bounded sampling frame was considered to be adequate for the study.

### Blood sample collection and species identification

Laboratory technologists working at the health centre collected venous blood after obtaining the research participants’ and or guardians’ consent. One millilitre venous blood was collected with single use sterile syringe and transferred into a tube that contains sodium citrate. Immediately about 2 µl of blood was used for the preparation of thin and 6 µl thick blood film. After air dry of thin smear first it was fixed by methanol and after thick blood film dried by air, the slide was stained in 10% Giemsa for about 10 min. At the end the slide was washed by clean water and air-dried. The laboratory technologists at the primary healthcare centre examined the slides as part of their regular health service provision. They checked for the presence or absence of parasites. After examining 200 white blood cells and not finding any parasites, the slide was declared negative. No specific training was provided to the technologists, and there were no specific quality assurance measures in place during the study.

Treatment was given to the positive cases according to national malaria treatment guidelines, if a patient was positive by microscopy. Three spots were prepared in each Whatmann™ 3MM (VWR®) filter paper. About 20 μl in each spot and a total of 60 μl of blood sample was spotted on one Whatmann™ 3MM (VWR®) filter paper for validation by nested nPCR. The blood on the filter paper was air dried to prepare dry blood spot (DBS). The dried DBS was placed in zip locked plastic bag with silica gel. Then, it was transported to Arba Minch University-Advanced Medical Entomology and Vector Control Laboratory for molecular analysis.

### *Plasmodium* parasites detection by nested PCR

#### DNA extraction from DBS

DNA extraction from DBS was done using the Saponin/Chelex Extraction method [12]. In brief, 6 mm DBS treated with saponin in phosphate buffered saline solution over night at 4 °C on a plate shaker. The final elute was done in 80 μl. DNA-was stored at − 80 °C until further use.

#### Identification of malaria parasites

*Plasmodium* species identification was done using the 18S rRNA nPCR according to the protocol by Snounou et al*.* [13]. In brief, genus-specific primers forward and reverse reactions for every single reaction were used. For N-1 amplification reaction at 1200 base pair cycling condition was set. After n-1 PCR amplification was completed, n-2 PCR reaction, *P. falciparum* and *P. vivax* species-specific primers were used. Both positive and negative controls were run together with the samples, thermo cycler using thermo cycler BIO RAD 100™ thermal cycler (Bio-Rad, Hercules, California, United States). At the end of second PCR amplification, agarose gel (Thermo Fisher Scientific Inc.) was prepared. Amplified products were run for gel-electrophoresis. Gel-red was used for staining. Amplified products were visualized as a band under a clear view of UV transilluminator and the results were recorded using micro doc cs scientific Ldt gel-doc. Presence of 205 bp band was considered as a sample was positive for *P. falciparum* and 120 bp band was considered as a sample was positive for *P. vivax.*

#### Data organization and analysis

Data collected from questionnaires and laboratory findings were entered into Microsoft Excel and analysed using SPSS version 20 software. Descriptive data were used to show the proportion of *Plasmodium* parasites detected by microscopy and nPCR, along with age, sex, and body temperature. A 2 × 2 contingency table computed sensitivity, specificity, and positive and negative predictive values. The Kappa (k) measure of inter-rater agreement was used to calculate the agreement between microscopy and nPCR.

## Results

### *Plasmodium* parasites diagnosis by microscopy and nPCR

**Fig. 1:**
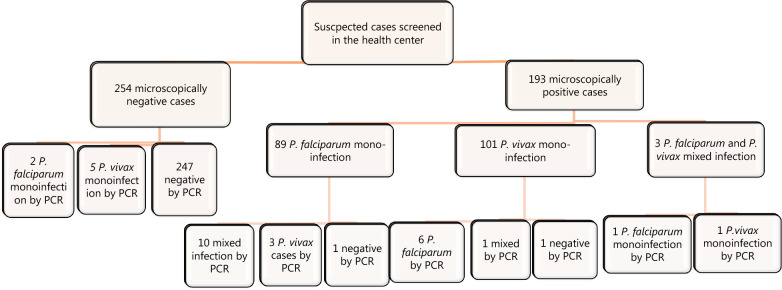
Flow diagram of malaria diagnosis by microscopy followed by nPCR confirmation

Of the 193 microscopically confirmed malaria positive cases, 46.1% (89/193) were due to *P. falciparum* mono-infection, 52.3% (101/193) were due to *P. vivax* and 1.6% (3/193) was *P. falciparum* and *P. vivax* mixed infection. Of the 89 *P. falciparum* mono-infection confirmed by microscopy, 11.2% (10/89) were *P. falciparum* and *P. vivax* mixed infection, 3.4% (3/89) were *P. vivax* and a single case was negative by nPCR. Furthermore, of the 101 microscopically diagnosed *P. vivax* mono-infections, 5.9% (6/101) were *P. falciparum*, 1% (1/101) were *P. vivax* and *P. falciparum* mixed infection and a single case was negative by nPCR. Of the 254 microscopically negative cases screened by nPCR, 0.8% (2/254) were positive for *P. falciparum* mono-infection and 2% (5/254) for *P. vivax* mono-infection. Of the three mixed infections identified microscopically one was found to be *P. falciparum*, one was *P. vivax* and one case was mixed infection by nPCR (Fig. 1).

By taking nPCR as confirmatory reference, the variation documented between microscopic diagnoses and nPCR was 12.4% (24/193) for positive cases and 2.8% (7/254) for negative cases (Fig. 1). Moreover, the variation documented between microscopic diagnoses of *P. falciparum* was 15% (14/89) compared with nPCR, and it was 7.9% (8/101) for *P. vivax*. Males accounted for 53.5% (239/447), with the median age of the study participants being 21 (range between 5 and 74 years) (Table 1).

**Table 1:** Malaria suspected cases, microscopically and PCR confirmed cases in Arba Minch zuria district, southwest Ethiopia (November 2019–Janurary 2020)

Variable	Microscopy results N (n, %)					PCR results N (n, %)				
	Pf	Pv	Mix	−ve	Total	Pf	Pv	Mix	−ve	Total
Sex										
Male	67 (28.0)	57 (23.8)	3 (1.3)	112 (46.9)	239	65 (27.2)	54 (22.6)	10 (4.2)	110 (46.0)	239
Female	22 (10.6)	44 (21.2)	0 (0)	142 (68.2)	208	19 (9.1)	48 (23.1)	2 (1)	139 (66.8)	208
Age										
5–14	24 (20)	49 (40.8)	2 (1.7)	45 (37.5)	120	20 (16.6)	51 (42.1)	5 (4.1)	45 (37.2)	121
≥ 15	65 (19.9)	52 (15.9)	1 (0.3)	209 (63.9)	327	64 (19.7)	51 (15.6)	7 (2.1)	204 (62.6)	326
Axillary temperature										
< 37.5	49 (13.9)	56 (17.3)	1 (0.3)	217 (67.2)	323	42 (13.1)	55 (17.1)	10 (3.1)	214 (66.7)	321
≥ 37.5	40 (32.3)	45 (36.3)	2 (1.6)	37 (29.8)	124	42 (33.3)	47 (37.3)	2 (1.6)	35 (27.8)	126

### Comparison of malaria microscopy versus nPCR technique

The sensitivity of microscopy in detection of *P. falciparum* was 89.3 (95% CI 86.2–92.8) and its specificity was 96.1 (95% CI 94.3–97.9) (Table 2). 94.8% agreement was reported between microscopy and nPCR with a strong agreement of the kappa value of 0.834. For *P. vivax*, the sensitivity of microscopy was 91.2 (95% CI 88.6–93.8) and the specificity was 97.7 (95% CI 96.3–99.1) with a kappa value of 0.89 using nPCR as a reference. Considering nPCR as a reference, the sensitivity of the microscope to detect *P. falciparum* and *P. vivax* mixed infection was low (8.3; 95% CI 5.7–10.9), but its specificity was 99.5 (95% CI 98.8–100).

**Table 2:** Performance of microscope in diagnosis of malaria compared to nPCR, Arba Minch Zuria district, Southwest Ethiopia (November 2019–Janurary 2020)

*Plasmodium* species	PCR		Total	Sensitivity (95% CI)	Specificity (95% CI)	PPV (95%CI)	NPV (95% CI)	Percentage agreement (%)	Kappa value
	+ve	−ve							
Microscopy vs. PCR									
Overall									
+ve	191	2	193	96.5 (94.7–98.3)	99.2 (98.4–100)	99.0 (98.1–99.9)	97.2 (95.6–98.8)	97.9	0.959
−ve	7	247	254						
*P. falciparum*									
+ve	75	14	89	89.3 (86.2–92.8)	96.1 (94.3–97.9)	84.3 (80.9–87.7)	97.5 (96.0–97.0)	94.8	0.834
−ve	9	349	358						
*P. vivax*									
+ve	93	8	101	91.2 (88.6–93.8)	97.7 (96.3–99.1)	92.1 (89.6–94.6)	97.4 (95.9–98.9)	96.2	0.89
−ve	9	337	346						
Mixed infection									
+ve	1	2	3	8.3 (5.7–10.9)	99.5 (98.8–100)	33.3 (28.9–37.7)	97.5 (96.0–99.0)	12.4	0.03
−ve	11	433	444						

+ve: positive; PPV: positive predictive value; −ve: negative; NPV: negative predictive value

The positive predictive value of microscopy compared with the nPCR in diagnosis of mixed infection was 33.3 (95% CI 28.9–37.7), but the negative predictive value was 97.5 (95% CI 96.0–99.0) (Table 2).

## Discussion

Many *P. falciparum* and *P. vivax* mixed infections have been microscopically overlooked and underreported in routine healthcare system. Using the molecular technique, 11.2% of *P. falciparum* and *P. vivax* mixed infections were identified among *P. falciparum* mono-infection confirmed microscopically. Additionally, *P. vivax* (3.4%) was misdiagnosed into *P. falciparum* and *P. falciparum* (5.9%) into *P. vivax* microscopically. A bout 2.8% of false negatives were documented microscopically and left untreated, which could have implications for malaria control and elimination.

Many mixed *P. falciparum* and *P. vivax* infections were microscopically incorrectly identified as *P. falciparum* mono-infection. Identifying the mixed infections of *P. falciparum* and *P. vivax* has been the long-standing problem in Ethiopia [14, 15]. In an earlier study, only 45% microscopists in hospitals and health centres accurately identified mixed infection of *P. falciparum* and *P. vivax* in Ethiopia [14], which needs an attention to improve malaria surveillance system for effective control and elimination [16]. Most mixed infections were identified from microscopically confirmed *P. falciparum* mono-infection, which might suggest that *P. falciparum* parasites are more dominant where they co-exist in the same patient. Hence, the person performing the microscopy might stop field examination after identifying the predominant parasite species [19, 20]. This might enforce the use of sensitive diagnostic methods and highlights the need to train malaria laboratory technicians to effectively diagnose [21]. Therefore, mixed *P. falciparum* and *P. vivax* infections are microscopically underestimated in the routine healthcare system. More sensitive diagnostic tools are needed to detect more cases of malaria. In this regard, Rapid Diagnostic Test (RDT) can improve malaria diagnosis due to its ease of use and independence from human skill [22]. However, RDT accuracy can also be compromised by HRP2/3 gene deletion and parasite density [23]. Although there are challenges, microscopy is still a reliable method to detect mono-infections.

Although identifying the two parasites involved in the mixed infection was the main obstacle, nPCR testing revealed that 2.8% of cases confirmed negative under microscopic examination were actually positive. Unfortunately, these cases were not treated within the healthcare system. False negative results can have an impact on malaria control and elimination because they prolong parasite transmission when untreated [8]. Additionally, failure to identify a second infecting parasite species in a mixed infection might result in the ineffectiveness of the therapy [15, 17]. Inadequate therapy can promote the spread of parasites and may prolong the duration of the illness since the parasite may not be cleared [9, 18]. To address this issue, regular checks should be conducted to identify gaps in the routine healthcare system for accurate malaria diagnosis. If this does not happen, the malaria elimination venture might be in jeopardy because untreated patients could continue transmitting and inadequate treatment could not be enough to get rid of the parasite and stop transmission swiftly.

The sensitivity and specificity of microscopy in detecting the mono-infections was over 89% using the molecular method as the reference. However, in the case of detecting mixed infections of *P. falciparum* and *P. vivax,* the sensitivity was very low as documented in the previous study [16]. This study is consistent with another study that emphasizes the challenge of using microscope-based diagnosis to detect mixed infections compared to nPCR [24]. The duration of time the laboratory technologists observing the parasites, as well as the relative density of the parasites in the mixed infections, might have contributed to this outcome.

This study has some limitations. The parasite density data was not included; therefore, assessing its impact on diagnosing microscopic malaria is necessary. Another limitation is the limited number of health facilities included; thus, increasing their number could improve representation and generalizability.

## Conclusion

Microscopy for malaria diagnosis may underestimate the number of mixed *P. falciparum* and *P. vivax* infections and misdiagnosed or missed cases of *P. falciparum* and *P. vivax*. While misdiagnosis is a concern, misidentifying *P. falciparum* as *P. vivax* would be more problematic. Based on the current findings, some cases were improperly treated or left untreated by the routine healthcare system. Therefore, the routine malaria diagnostics system could be monitored regularly, and tailored action should be taken to minimize this problem.

## Data Availability

All data for this study will be available upon request.
